# Melioidosis clinically unresponsive to meropenem in a traveller returning from Thailand

**DOI:** 10.1016/j.idcr.2025.e02407

**Published:** 2025-10-22

**Authors:** Ilka Grewe, Anette Hennigs, Flaminia Olearo, Marylyn M. Addo, Michael Ramharter, Sabine Jordan

**Affiliations:** a I Department of Internal Medicine, Division of Infectious Diseases, University Medical Center Hamburg-Eppendorf, Martinistraße 52, Hamburg 20251, Germany; b Institute for Infection Research and Vaccine Development (IIRVD), Center for Internal Medicine, University Medical Center Hamburg-Eppendorf, Hamburg, Germany; c Department for Clinical Immunology of Infectious Diseases, Bernhard Nocht Institute for Tropical Medicine, Hamburg, Germany; d German Center for Infection Research, Partner Site Hamburg-Lübeck-Borstel-Riems, Hamburg, Germany; e Center for Diagnostics, Institute of Medical Microbiology, Virology and Hygiene, University Medical Center, Hamburg-Eppendorf, Hamburg, Germany; f Department of Tropical Medicine, Bernhard Nocht Institute for Tropical Medicine, Hamburg, Germany

**Keywords:** Melioidosis, *Burkholderia pseudomallei*, Antibiotic resistance, Carbapenem resistance

## Abstract

*Burkholderia pseudomallei* can cause severe systemic infection with involvement of multiple organs and abscess formation. Carbapenems are considered superior to ceftazidime in severe cases.

We here report a case of Melioidosis in a previously healthy 57-year-old traveller. Despite *in vitro* testing confirming susceptibility of the isolate for meropenem, the patient’s clinical condition did not improve during seven days of meropenem treatment. After changing the treatment regimen to high-dose ceftazidime, the patient’s clinical condition and laboratory parameters rapidly improved. Thus, we here report a rare case of Melioidosis clinically unresponsive to meropenem.

## Introduction

Melioidosis is a common cause of pneumonia and septicaemia in South-East-Asia and Australia, but is rarely diagnosed in travellers returning from endemic areas [1]. Clinically, the infection can cause abscess formation and multiple organ involvement. Risk factors for a severe disease course include diabetes mellitus, chronic lung diseases and chronic kidney injury [2]. *Burkholderia pseudomallei*, the gram-negative bacteria causing melioidosis, is naturally resistant to a range of antibiotics. Resistance occasionally evolves during antibiotic treatment, but since *B. pseudomallei* is not spread from human-to-human resistant strains are not rapidly spread. Treatment includes an intensive intravenous antibiotic regimen during the acute phase, followed by an oral eradication therapy to prevent relapse [3], [4]. High-dose ceftazidime has shown efficacy for initial therapy during the acute phase in a randomized clinical trial and may be used for non-critically-ill patients [5]. Since observational studies point towards superiority of carbapenems compared to ceftazidime, to date meropenem is considered to be the gold-standard for severe infections [6], [7], [8], [9]. However, prospective clinical studies comparing carbapenem and cephalosporine therapy for melioidosis are scarce [10]. Carbapenem resistances have been described to evolve during therapy [11]. Here, we describe a case of severe infection with *B. pseudomallei*, that did not improve under anti-infective treatment with meropenem, but responded well to ceftazidime.

## Case

**Fig. 1:**
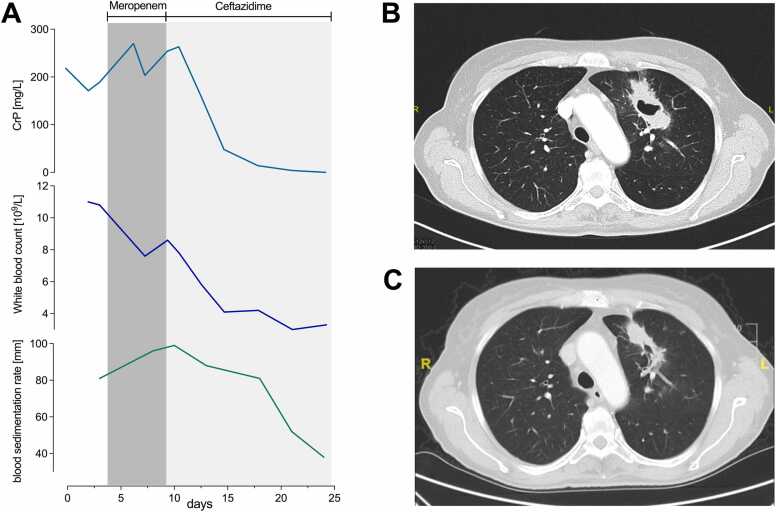
Time line including CrP [mg/L], white blood count [10^9^/L] and blood sedimentation rate [mm] during meropenem and ceftazidime treatment (A). Computertomography before effective anti-infective treatment (B) and after 14 days of ceftazidime treatment (C).

A 57-year-old female with no known pre-existing condition presented to our outpatient clinic with fever up to 39,7 °C, headache and dyspnea. One week prior to consultation she had returned from a seven-week travel to Thailand and Cambodia, where she spent majority of her time in Bangkok, Phuket, Koh Samui and Siem Reap. Laboratory results revealed CrP-elevation of 218 mg/L, white blood count of 11.0 × 10^9^/L and an increased blood sedimentation rate of 81 mm (Fig. 1A). A chest X-ray showed a consolidation in the left upper lung. An empirically initiated treatment with amoxicillin and clavulanic acid (875/125 mg orally every eight hours) for suspected community-acquired pneumonia did not lead to improvement of the patient’s clinical condition. The radiographic finding in the chest X-ray presented as a solid consolidation with a caverna in a consecutive CT-scan (Fig. 1B). Since the patient’s condition continuously worsened, including progressive dyspnea and somnolence, she was admitted to our clinic and antibiotic therapy was escalated to meropenem (1000 mg intravenously every eight hours). Notably, fever, dyspnea and headache continuously worsened and CrP increased under meropenem treatment (Fig. 1A). In a bronchoalveolar lavage *Burkholderia pseudomallei* was identified via culture and subsequent Matrix-assisted laser desorption ionization–time of flight mass spectrometry (MALDI-TOF). Although antimicrobial susceptibility testing showed the isolate to be susceptible to meropenem with a Minimum Inhibitory Concentration (MIC) of 1 μg/mL, the patient’s condition did not improve during seven days of meropenem treatment. Therefore, antibiotic treatment was changed to high-dose ceftazidime (2000 mg intravenously every six hours), for which the isolate was also tested susceptible with a MIC of 1 μg/mL. Subsequently, the patient’s clinical condition rapidly improved and laboratory parameters normalized. A control CT-scan showed regression of the cavernous consolidation in the left upper lung (Fig. 1C). Following 14 days of intravenous ceftazidime, oral cotrimoxazole/trimethoprim was initiated during which the patient experienced a relapse of fever. Eradication therapy was therefore switched to doxycycline, which was administered for six months, resulting in full recovery.

## Discussion

*B. pseudomallei* is highly endemic in Southeast Asia and northern Australia, where *B. pseudomallei* is commonly found in soil and water [7]. While most severe cases of Melioidosis occur in patients with chronic conditions such as chronic lung disease, diabetes mellitus or chronic kidney injury, we here present a severe case of Melioidosis in a previously healthy traveller returning from Thailand to Germany [12], [13].

Natural resistances of *B. pseudomallei* towards carbapenems have not been reported previously, but carbapenem resistances occasionally evolve during treatment [11], [14]. *In vitro* susceptibility testing showed that the vast majority of isolates in Thailand were sensitive to meropenem (98 %), along with high susceptibility rates to ceftazidime [15]. Epidemiological data on clinical responsiveness to antibiotic treatment in the area are scarce. Also globally, meropenem resistance is extremely rare in patients without previous meropenem exposure [16], [17]. Since our patient did not have any history of previous carbapenem treatment, this case represents a rare case of Melioidosis that did not respond to initial treatment with meropenem.

Previous studies showed superiority of carbapenems compared to ceftazidime in treatment of severe melioidosis [10], [18]. This case emphasises that in carbapenem unresponsive cases, a treatment regimen with high-dose ceftazidime can be considered. *In vitro* susceptibility testing did not confirm meropenem-resistance. Nevertheless, disease severity continuously worsened during seven days of meropenem treatment and rapidly mitigated after change to a high-dose ceftazidime regimen. The reason for the discrepancy between in vitro antimicrobial resistance testing and clinical treatment failure remains unclear. A previous study reported growth-defective *B. pseudomallei* associated with ceftazidime treatment failure, leading to a lack of *in vitro* detection of resistant isolates [19]. This phenomenon has not been described for meropenem-resistant isolates yet.

A key contributor to the carbapenem resistance in *B. pseudomallei* is the active efflux of antibiotics mediated by resistance-nodulation-cell-division (RND) efflux pumps [11], [20]. Upregulation of expression of efflux pumps usually occurs during antibiotic treatment and is only rarely observed in initial isolates. While this may represent a possible underlying mechanism for the observed clinical treatment failure, gene expression of efflux pumps was unfortunately not assessed in the present case. Furthermore, mutations upstream of the *penA* gene, leading to an overexpression of class A β-lactamase can result in ceftazidime resistance and also in decreased meropenem sensitivity [21]. Given the patient’s clinical response to ceftazidime, a *penA*-mediated mechanism of resistance appears unlikely in this case. Due to the single-case-design of the study, no further direct conclusions on antibiotic therapy can be drawn. However, this case emphasises the need for awareness and further investigation on carbapenem unresponsive Melioidosis.

## Conclusion

We here report treatment failure of meropenem as initial therapy for a severe case of *B. pseudomallei* infection. Although resistance was not confirmed microbiologically, clinical symptoms and laboratory results reflect a better response to ceftazidime. Thus, this is a rare case in which ceftazidime treatment was superior to meropenem.

## CRediT authorship contribution statement

**Sabine Jordan:** Writing – review & editing, Supervision, Investigation, Conceptualization. **Ilka Grewe:** Writing – review & editing, Writing – original draft, Visualization, Investigation, Conceptualization. **Flaminia Olearo:** Writing – review & editing, Investigation. **Anette Hennigs:** Writing – review & editing, Validation, Supervision, Conceptualization. **Michael Ramharter:** Writing – review & editing. **Marylyn M. Addo:** Writing – review & editing, Conceptualization.

## Ethical approval

The study was conducted in accordance with the local legislation and institutional requirements. The patient granted permission to publish her medical history.

## Patient consent

The patient granted permission to publish her medical history.

## Funding

IG was supported by the iDfellows program (493624519) funded by the 10.13039/100004807DFG.

## Conflict of Interest

The authors have declared no conflicts of interest.
